# Face-to-Face Clinical Practice Under COVID-19 Pandemic: How Psychotherapists Describe Their Experiences

**DOI:** 10.3389/fpsyg.2021.726439

**Published:** 2021-08-12

**Authors:** Eugénia Ribeiro, Ângela Ferreira, Cátia Cardoso, Raquel Queiroz, Vânia Silva

**Affiliations:** Psychotherapy and Psychopathology Research Lab, CIPsi, School of Psychology, University of Minho, Braga, Portugal

**Keywords:** face-to-face psychotherapy, face masks, COVID-19 pandemic, verbal and non-verbal communication, therapeutic relationship

## Abstract

Driven by the theory-building around the role of the non-verbal components to communication, we aimed to understand how therapists experience the therapeutic process using a facial mask. The empirical evidence of the power of non-verbal communication to engage therapists and clients in therapeutic work, develop a positive and collaborative relationship between them, and display empathy is quite large. A mixed-methods approach was adopted, drawing from the therapists’ participation in an online survey. A sample of 137 psychotherapists with different therapy orientations and years of clinical practice participated in the study. Therapists conducted face-to-face therapy wearing face masks with existing and/or new clients. We performed an exploratory analysis, using descriptive statistics, to explore the psychotherapists’ evaluations regarding perceived impact of face masks on different therapy quality dimensions. In a complementary rationale, we analyzed the therapists’ perspectives on their experience wearing face masks using the thematic analysis methodology. Results show that among 137 psychotherapists, 114 were attending both existing and new clients, whereas only 13 were seeing exclusively existing clients and 10 were working exclusively with new clients. Despite no major differences were found between conditions regarding the perceived impact of face masks on different therapy quality dimensions and strategies adopted, the qualitative analysis allowed us to expand the quantitative results and deepen understanding of psychotherapists’ experience. Based on general and typical patterns, we propose two distinct models to describe the therapist’s experiences narrative when working with existing or new clients wearing face masks. Based on the results, we propose some recommendations to clinical practice in similar conditions.

## Introduction

With the outbreak of the COVID-19 pandemic, several public health measures were gradually implemented to avoid the transmission of the SARScov2. The mandatory use of face masks in public settings in many countries has brought sudden changes to daily life. Healthcare settings, including face-to-face mental health care, have been challenged. It seems that wearing face masks has a significant impact on verbal and non-verbal communication, affecting in turn the quality of the therapeutic relationship, in general, and their specific elements such as therapeutic alliance and therapeutic collaboration.

Empirical evidence to the therapeutic alliance as a mechanism that prompts change has been well documented in the literature by several meta-analyses, supporting its predicted value to therapeutic outcomes (e.g., Horvath et al., 2011; Flückiger et al., 2012, 2018). Despite the main focus on the verbal content of the therapists and clients’ interactions as an indicator of the therapeutic alliance quality, the non-verbal aspects of the psychotherapy process have gotten attention in the research field some time ago. In a research overview about the non-verbal behavior in therapist and client interactions, Hall et al. (1995) highlighted the role of the therapist’s moderate head nodding and smiling, frequent eye contact, active facial responsiveness, and a warm and relaxed tone of voice to engage clients on the therapeutic work.

Regarding the clients’ non-verbal behavior, the earliest studies were mainly focused on non-verbal cues to assess psychopathology and help therapists to define the therapeutic goals. More recent studies about the impact of non-verbal behaviors in psychotherapy state the influence of these behaviors on client’s satisfaction with therapy, treatment adherence, and therapeutic outcomes. These studies (Roter et al., 2006; Foley and Gentile, 2010; Del Giacco et al., 2020) have shown that the quality of the therapeutic alliance is influenced by the therapist’s and client’s non-verbal modes and behaviors, which carry important information about the emotional experience of both elements of the dyad and the client’s engagement and confidence. Thus, the information provided by non-verbal behaviors can be used to establish rapport between client and therapist and guide psychotherapy in a tolerable and therapeutic way toward mutual goals (Foley and Gentile, 2010). In a recent study with depressed patients, Del Giacco et al. (2020) confirmed that the collaborative behaviors that lead to the construction of the therapeutic alliance are determined not only by the therapist’s and client’s verbal modes but also non-verbal modes, like an elaborative vocal mode employed by therapists and an emotional vocal mode by clients.

According to the Basic Emotion Theory (BET) core assumptions, emotions are the “grammar of social living” (Keltner et al., 2019, p.133); that is, the emotional expressions coordinate the interactions within meaningful relationships, as is the case of therapeutic relationships. Consistently with the BET assumptions, the last decades of research on the area indicate that emotional expression is a multimodal and dynamic pattern of behavior. These patterns involve different modalities such as facial action, vocalization, bodily movements, gaze, gestures, head movements, touch (e.g., De Gelder et al., 2006; App et al., 2011; Krumhuber et al., 2013; Keltner et al., 2019; Papapicco et al., 2021). In reviewing the empirical literature on emotional expression and perception, Keltner et al. (2019) affirmed that some studies have contributed to differentiate expression of a broader range of emotions beyond the basic six emotions early studied, and upward of 20 emotions were found to have distinct multimodal expression. Relevant to our study is to note that almost all the 24 emotional states studied (e.g., sadness, anger, boredom, fear, and pain) were reliably communicated by multimodal patterns involving facial and vocal modalities. In addition, studies have shown that emotional expression provides valuable information to perceivers to guide their consequent behavior, evoke specific responses in social perceivers, signal the sender’s trustworthiness, and influence the dynamic and structure of the interaction. For example, in a review of the effects of dynamic aspects of facial expressions, Krumhuber et al. (2013) found that people trust interlocutors and provide more information if they observe authentic smiles (which have more prolonged onset and offset times). The same author concluded that in addition to the multimodal nature, the dynamic information of emotional expression (direction, quality, and speed of motion) significantly impacts the emotion perception quality and accuracy.

On the other hand, Sauter et al. (2010) focused on acoustic cues to identify ways in which emotions can be expressed non-verbally, like laughers and screams. They found that most basic emotions can be predicted by affective vocalizations ratings, similar to facial affective signals. The authors highlighted that emotionality expressed in the face and inflected in the speech provides perceptual cues to recognize positive and negative non-verbal expressions of emotion accurately.

In a complementary line of research, several empirical studies have found that features of social and cultural contexts shape emotion expression and perception (e.g., Elfenbein et al., 2007; Barrett and Kensinger, 2010; App et al., 2011; Keltner et al., 2019; Papapicco et al., 2021). For example, a recent study that analyzed micro-facial expressions as indicators of the emotional experience of brain drain during narrative interviews found that the emotional experience as inferred by facial expression seems to be gender and the acculturation process-dependent (Papapicco et al., 2021).

In a critical review of the literature about facial movements typically called facial or emotional expression, Barrett et al. (2019) emphasized the variations in the facial configurations and movements specific to an expression of each emotion category. The same authors highlighted the context-dependent nature of facial movements, proposing to be influenced by the immediate and outward context of the person expressing the emotion. When applied to therapy, the immediate context could be the internal state or the activated past experiences. The outward context could be both therapist and clients’ culture and the therapeutic setting condition (e.g., wearing a face mask).

Therefore, the role of emotional expressions and perceptions in social and meaningful interactions is particularly relevant in psychotherapy, where a trustful and respectful relationship is the basis of the therapeutic work. The use of face masks, a key strategy to prevent the spread of SARScov2, seems to significantly impact verbal and non-verbal communication, which may disturb the multimodal and dynamic expression and perception of emotions.

Recent studies (Carbon, 2020; Marler and Ditton, 2020) showed that mask wearing affects social interaction while disturbing emotion reading from facial expressions. Carbon (2020) found that many emotional states such as happy, sad, and angry were misinterpreted as neutral, and emotions such as disgusted were confused with angry. According to Marler and Ditton (2020), people infer emotions based on expression cues displayed by the mouth and, thus, covering the mouth may have a significant impact on non-verbal communication. The participants from a study carried out by Saunders et al. (2020) described that, besides making communication fatiguing, frustrating, and embarrassing, face masks wearing affect the communication content, interpersonal connection, and the disposition to engage in conversation. Since it compromises peoples’ connectivity through facial expression, it may have negative psychological effects, leading to the dehumanization of social relationships (Vainshelboim, 2020).

Similarly, in healthcare settings, face masks create a physical barrier that may interfere with the ability to successfully communicate and, thus, affect the collaborative process and the establishment of the therapeutic alliance between clinician and patient. Therapists rely on non-verbal communication as facial expression and body language to convey meaning and designate reassurance, affirmation, and empathy (Marler and Ditton, 2020). Wong et al. (2013) found that the use of face masks by medical doctors during consultations had a significant negative impact on the patient’s perceived empathy.

### Purpose of the Present Study

Given the scarcity of prior studies on the therapists’ experiences during the COVID-19 pandemic and the theoretical and empirical framework explained above, it is crucial to study the impact and experience of wearing face masks in psychotherapy. As some non-verbal cues, facial configuration, or some facial movements are no longer available or can be incomplete with the use of face masks, therapists have been challenged to find other ways to build a connection with their patients.

Thus, considering individual psychological intervention with adults during the COVID-19 pandemic, this study aimed to analyze how psychotherapists in Portugal experience the face-to-face therapeutic process wearing a face mask and to identify possible strategies to manage the challenge of this experience. Following this general aim, we proceeded with two specific aims. First, we aimed to investigate the perceived impact of the face mask on the quality of the session, therapeutic alliance, communication, emotional and safety experiences, as evaluated by therapists in a questionnaire developed and based on the empirical literature. In accordance with literature findings, we expected that therapists evaluated negatively the impact of wearing face masks on the therapeutic relationship and communication. Furthermore, we expected that this negative evaluation was worse with reference to the therapy with new clients when compared with therapy with existing clients. Second, we aimed to understand the therapists’ perspectives about their own experience of wearing face masks in the therapy.

To date, two *Emergency States* were implemented in Portugal to contain the pandemic: the first one began on March 19, 2020, and it was extended until May 2, 2020 (Decree-law no. 14-A/2020, 2020); the second one started on November 6, 2020, and it was extended until May 1, 2021 (Decree-law no. 51-U/2020, 2020). Driven by the uncertainty of the COVID-19 pandemic’s evolution, this study is relevant to understand the impact of face mask wearing on face-to-face therapeutic processes and to identify practice-based recommendations on how to take therapeutic and productive action in likely scenarios.

## Materials and Methods

### Design and Sample

This exploratory study followed a mixed-methods approach by combining quantitative and qualitative data from therapists’ responses to an online survey questionnaire. We cross-analyzed the data to explore in depth our phenomena of interest. By analyzing the therapists’ answers to the closed-ended questions, we reached the first aim (to investigate the perceived impact of the face mask on the therapy process). By examining the therapists’ responses to the open-ended questions, we realized the second aim (understanding therapists’ perspectives about their own experience).

Eligible participants included all licensed psychologists, namely clinical psychologists and psychotherapists, who are registered into the Portuguese Psychologists Association [Ordem dos Psicólogos Portugueses (OPP)]. It is the only organization responsible for the professional title in Portugal (Decree-law no. 57/2008, 2008) and membership has been mandatory since 2008. According to the latest OPP census (2014), there are 12732 registered professionals, and it is estimated that nearly 51% are working in the field of clinical psychology (Coelho and Brás, 2012).

A purposive sampling method was employed to recruit potential participants on social media platforms (e.g., Facebook and LinkedIn), as well as on the main directory of psychotherapy associations and societies in Portugal (available on the OPP site). All participants who provided a valid e-mail address were contacted by the authors. An external link that led to the online survey was sent, where interested participants could read further information regarding study objectives and inclusion criteria.

The selection criteria for survey participants required them to be a licensed psychologist (i.e., being registered at OPP) with current clinical practice and have experience in face-to-face modality regarding individual psychological intervention with adults during the COVID-19 pandemic.

To start the survey, participants had to agree to the data protection declaration (i.e., informed consent form). No incentives were provided, and participation was voluntary. The ethics committee of the University of Minho approved the study [CEICSH 023/2021]. The entire procedure lasted approximately 15 min. Data were collected from the 22nd of February until the 21st of March.

### Survey Development and Design

An online survey was created for this study using the Google Forms system. Its content was derived from the authors’ review of relevant literature and discussion with colleagues. A preliminary version was created, pilot tested with three participants, and subsequently checked by the authors. Changes were made in line with suggestions emerging from this process. Additionally, we added screening questions to prevent individuals who do not meet the target criteria from taking our survey to get valid data. For some questions, we delimited the possibilities of the answer to avoid unrealistic responses (e.g., set the age range from 18 to 99). To avoid bias in the responses, we included inverted items in the survey. The final version of this online survey focused on a number of key issues, which included:

(1) Information relating to participant characteristics (i.e., sex, age, nationality, educational level, years of clinical experience, and psychotherapeutic orientations).
(2) Information relating to individual psychological intervention since the COVID-19 pandemic outbreak (i.e., modalities being used by psychotherapists; client types–whether they were new, existing, or both, number of clients treated on average per week in personal contact, clients’ disorders, and adopted protective measures against COVID-19).
(3) Overall experience of the face-to-face therapeutic process using a face mask.
(4) Perceived impact of wearing a face mask on session quality, therapeutic alliance, verbal and non-verbal communication, and emotional and safety experiences.
(5) Perceived positive and negative aspects of face-to-face modality during COVID-19.
(6) Psychotherapists’ recommendations on how to manage a face-to-face setting during the ongoing COVID-19 pandemic.

After completing survey items regarding demographic characteristics and professional background (sections 1 and 2 of the survey), participants were asked about the frequency they used different modalities during the COVID-19 lockdown, namely: face-to-face, telephone, messaging, emails and video-link. A five-point Likert scale was developed where the following values were assigned to each response (1 = “never,” 2 = “rarely,” 3 = “often,” 4 = “always,” and 5 = “exclusively”). Participants were also asked to rate the frequency they can adopt four protective measures against COVID-19 on a four-point response scale (1 = “never,” 2 = “rarely,” 3 = “often,” and 4 = “always”). The four protective measures, suggested by the Portuguese Directorate-General of Health (2020), are: (1) wear a facial mask; (2) maintain a physical distance of at least 2 m from each other; (3) wear a face shield; and (4) use physical barriers (e.g., transparent partitions or unidirectional mirror).

Moreover, each participant was asked the following open-ended question: “Considering the ongoing COVID-19 pandemic, how have you experienced the face-to-face therapeutic process wearing a face mask in individual psychological intervention with adults?” (section 3 of the survey).

Moving to section 4 of the survey, of the survey, participants were asked to rate the impact of wearing face masks on the quality of the session, therapeutic alliance, communication, emotional and safety experiences. This section includes 20 items, evaluated in the range of the five-point response scale from strongly disagreed (1) to totally agree (5). Examples of these items are: “Item 1. The session quality is good”; “Item 2. I can express myself and communicate clearly to the client”; “Item 6. I can clearly understand the client’s emotional experience”; “Item 12. I notice that the client feels safe in the therapeutic relationship”; and “Item 19. I feel at risk of being infected by the new coronavirus (SARS-CoV-2).”

In section 5 of the survey, we aimed to analyze which strategies the psychotherapists adopted in their face-to-face practice under the COVID-19 pandemic. The options of answers were yes or no and examples of these items are: “I speak openly with my client about the experience of using a face mask”; “I speak louder”; “I frequently make sure/ask to the client if I correctly understand him/her”; and, “I ask the client more often how he/she is feeling.” Additionally, psychotherapists were asked the following open-ended question: “What tips could you give to a colleague who is treating clients face-to-face and using a face mask for the first time?” (section 6 of the survey). Finally, participants were asked to identify two positive and two negative aspects of face-to-face modality during the COVID-19 pandemic (section 7 of the survey).

### Participants

**FIGURE 1:**
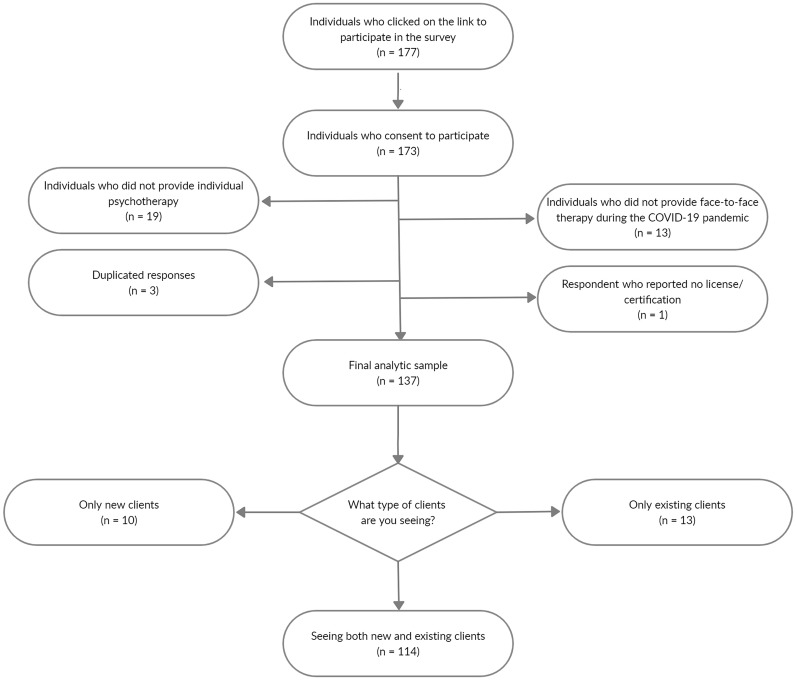
Flow chart of participants recruitment, attrition, and inclusion.

Interested participants were sent an invitation to click on an external link that led to the online survey, where they could read further information regarding study objectives and inclusion criteria. A total of 177 psychotherapists clicked on the link to participate in the survey, and 173 consented to participate (see Figure 1). For the present analyses, we excluded 13 participants who did not provide face-to-face therapy and 19 participants who did not provide individual psychotherapy in the outpatient treatment of adults under the COVID-19 pandemic. We additionally excluded three duplicated responses and one participant who are not registered on OPP. The final analytic sample included 137 participants with at least some experience in face-to-face practice under the COVID-19 pandemic.

### Analytical Strategy

#### Quantitative Data Analysis

We performed an exploratory analysis using descriptive statistics (e.g., percentages and means). The quantitative data analyses were conducted by the second author, using the statistical R software (R Core Team, 2021). Thus, as a first step, we described the sociodemographic characteristics of participants. Then, we analyzed the impact of wearing face masks on the quality of the session, dyad communication, quality of therapeutic alliance, emotional and safety experiences, as well as the strategies used by psychotherapists, for both new and existing clients, regarding face-to-face intervention.

#### Qualitative Data Analysis

In the current study, we conducted a thematic analysis using the procedures recommended by Braun and Clarke (2006), intended to make sense of data collected from participants’ responses to open questions. The analysis moved beyond the responses of the participants to identify patterns of meanings and develop semantic themes that organize the global meaning of the therapist’s experience when doing face-to-face therapy using a face mask.

First, we used an inductive analysis, driven by participant data, considering that the participant’s response to each interview question contributes to understanding the therapist’s experience globally. This analysis was done based on two groups of participants’ responses: therapy with existing clients and therapy with new clients. Based on this inductive analysis we construct a codebook, including themes and subthemes and then we coded the remaining data by deductive content analysis. The participants’ answers were analyzed in Portuguese, and the excerpts used to illustrate our results in the current paper were translated into English.

##### Thematic inductive analysis

At the first step, all researchers read the entire body of data, becoming familiar with participants’ responses to different open questions, making some brief notes, and registering general impressions about the data set. Based on this reading, the research team agreed to exclude all the responses that appear too vague and difficult to interpret the explicit meaning. For example, responses like “ethics” (to the question about recommendations to conduct face-to-face therapy using a face mask) or “incomplete” (in response to the question about therapist experience) were excluded.

In the second step, we aimed to generate initial codes. We labeled as the analytic unit each participant’s response that was considered to communicate meaning on the therapist’s experience and significant to the research scope. Coding was exhaustive and not exclusive; thus, each analytic unit was coded with as many initial codes (topic/meaning idea) as possible having in mind the context of the therapist’s experience. This step of generating initial codes was independently done by the first and fifth authors who developed codes using open coding based on responses of 100 therapists (50 of the condition “therapists with existing clients” and 50 of the condition “therapists with new clients). These initial codes were modified and revised by the team’s collaborative consensus. The research team met to discuss the initial code’s descriptions and disagreements on it, and the final codes were consensually adjusted and defined. We did this coding procedure by hand and worked with Microsoft Excel to organize codes. At the end of this second step, we came up with a list of 97 initial codes (based on 389 responses of 100 participants).

At the third step, intending to search for themes, codes were gathered and compared to one another having in mind the scope of the study. Based upon commonalities identified between codes we developed higher-level categories that formed initial themes. The first and the fifth author independently developed the main themes and subthemes supported by a preliminary thematic map that was based on the grounded relations between codes and themes and on memos registered during analysis (e.g., participants referred to difficulties but identified resources to solve them). After, the research team met to discuss and adjust the themes and subthemes based on the consensual process. This process continued until main themes and subthemes were stabilized.

In this fourth step, the third, fourth, and fifth researchers checked the main themes alongside the participants’ responses to decide that they communicated a reliable story of the therapists’ experiences as reported on data and answered the research question. This procedure allows us to refine some initial codes and main themes.

Finally, in this fifth step, the all-research team developed an analysis of each theme and consensually defined them, and decided their final names having in mind the grounding of their meanings and the immediate understanding of the relation between their designation and the scope of the study.

##### Deductive content analysis

A content analysis of responses from an additional 77 (1st condition) and 74 (2nd condition) participants was conducted in a similar way to the thematic analysis (based on the explicit meaning). However, as the themes and sub-themes included in the codebook already had reached saturation and had stabilized, the new analytic units (each response) were compared directly with the sub-themes and themes according to the codebook. An auditing check was done by the first author. Through this content analysis, no new sub-themes were generated as all the data coded fit into the existing sub-themes/themes and their designations did not change. Thus, this analysis confirmed the saturation of data analysis. This procedure allows us to combine an intensive thematic analysis to analyze the complete collected data.

##### Analysis of themes prevalence

We classified the prevalence of the themes and respective sub-themes as general, typical, variant, and rare, following the procedure suggested by Hill et al. (2005) and the recommendations for larger samples (Knox et al., 2006). Thus, we defined the prevalence based on the number of participants that contributed to the presence of each theme and sub-theme. We considered as *general* the themes that were present in data for ≥90% of participants, as *typical* the themes that were present in data for at least 50% until less than 90% of participants, as *variant* the themes that were present in data for at least 20% until 50% of the participants, and as *rare* the themes that were present in data for ≤20% of the participants.

##### Researchers

The first author is a clinical psychology researcher, and she has expertise in qualitative methods in psychology. As a psychotherapist, she uses cognitive behavior and constructivist approaches in her clinical practice. She is the head researcher of a therapeutic relationship research group. The second, third, and fifth authors have clinical training and practice in cognitive behavior therapy and the fourth author has training and practice in dynamic therapy. All the authors are members of the same research group and share research interests on the therapeutic relationship and dyadic processes as therapeutic alliance, therapeutic collaboration, and responsiveness. Being aware of their potential subjective influence, all authors discussed and reflected on their expectations regarding the research phenomena and worked to minimize their bias within the analytic process.

##### Methodological integrity

As recommended in the qualitative research literature (e.g., Levitt et al., 2016, 2018), we used different procedures to assure the integrity and credibility of the study. The researchers’ consensus processes through the different steps of inductive thematic analysis and the auditing check through content analysis contribute to enhance the trustworthiness of the findings in this study. In addition, we addressed the confirmability criteria by defining and illustrating the main themes and their sub-themes with participants’ representative quotations.

## Results

### Therapists and Therapy Characteristics

Some indication of how representative the sample might be of the wider clinical practitioner body comes from the sex breakdown of participants which was 117 (85%) female, 19 (14%) male, and 1 (1%) preferred not to say. These values correspond very closely to the sex breakdown reported in the last OPP membership survey–84% female and 16% male (Ordem dos Psicólogos Portugueses, 2014). Also, the mean age of participants was 42 (SD = 10) years, which is similar to the mean age reported by OPP survey–nearly 38 years.

Regarding educational level, almost half (47%) of the participants have an undergraduate degree (at least 5 years), 35% have a master’s degree, 17% are doctorate holders, and 1% have a post-graduate degree. All participants were Portuguese citizens and the average year in clinical practice was 14 (SD = 10). When inquired about their therapeutic orientations, the vast majority of psychotherapists identified themselves as cognitive-behavioral (32%), followed by those who have found themselves as psychodynamic/psychoanalytic (19%), or humanistic/experiential (18%). Other modality groupings were systemic or family therapy (10%), constructivist (9%), interpersonal (5%), eye movement desensitization and reprocessing (3%), and integrative (2%). Finally, an “other” category (2%) included art therapy, transpersonal, Eriksonian, and dialogical approach.

Psychotherapists were asked about the frequency they had been using different therapy modalities with clients during the COVID-19 lockdown. The use of face-to-face and video links were by far the most frequently used (86 and 82%, respectively). Conversely, in spite of lockdown context, the other types of remote working, that is telephone, messaging, emails were rarely or never used by the majority of participants (83, 93, and 93%, respectively).

Regarding the face-to-face contact after the outbreak of the COVID-19 pandemic, psychotherapists were also asked whether (a) they had seen clients previously, (b) they were only seeing new clients, or (c) they were seeing both new and existing clients. From Figure 1, it is clear that most psychotherapists were attending both types of clients (*N* = 114). Of the 137 participants in the present study, only 13 were seeing exclusively existing clients, and 10 were working exclusively with new clients. Furthermore, the vast majority of psychotherapists (45%) reported being treating, on average, less than six clients per week in personal contact, while 25% reported being treating from 6 to 10 clients. Of the remaining participants, 12% reported to be treating between 11 and 15 clients on average per week, and 18% reported to be treating 16 or more.

In terms of the client’s diagnosis, therapists are seeing more clients with anxiety (*N* = 138) and depression (*N* = 129) since the start of the coronavirus pandemic. Other disorder groupings with significant expression in demand were personality disorders (*N* = 56), obsessive-compulsive and related disorders (OCD; *N* = 49), substance-related and addictive disorders (*N* = 28), eating disorders (*N* = 28), schizophrenia and other psychotic disorders (*N* = 17), other conditions that may be a focus of clinical attention (*N* = 11), neurocognitive disorders (*N* = 5), and trauma and stressor-related disorders (PTSD; *N* = 1).

When asked how well they can adhere to the four protective measures during face-to-face psychotherapy, most of the participants indicated that they were always or often wearing facial masks (84 and 13%, respectively) and maintaining a physical distance of at least 2 m (73 and 22%, respectively). Conversely, only a small number of participants indicated that they were wearing a face shield (2% for always or often) or adopted other types of physical barriers such as transparent partitions or unidirectional mirrors (11% for always or often).

### Evaluation of the Perceived Impact of Wearing Masks on Therapy

**FIGURE 2:**
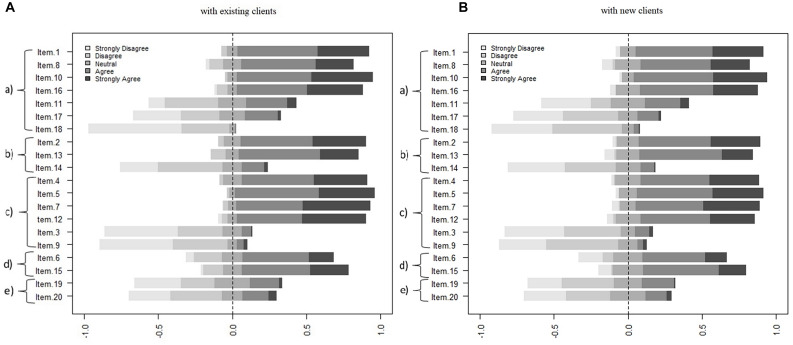
The perceived impact of wearing a face mask on five therapy dimensions. Panel **(A)** with existing clients. Panel **(B)** with new clients. (a) The quality of the session, (b) verbal and non-verbal communication, (c) therapeutic alliance, (d) emotional experience, and (e) safety experience.

Here we present the results about the therapists’ perceived impact of wearing masks on (a) the quality of the session, (b) verbal and non-verbal communication, (c) therapeutic alliance, (d) emotional experience, and (e) safety experience. As shown in Figure 2, for both conditions–attending existing clients vs. new clients–therapists rated similarly the impact of wearing a face mask on the five dimensions previously mentioned. Namely, with respect to (a) the quality of the session, for both conditions (i.e., existing clients vs. new clients), the vast majority of therapists agree or strongly agree with the following statements: “Item 1. The session quality is good” (89 and 86%, respectively); “Item 8. I feel comfortable in the session” (76 and 74%, respectively); “Item 10. The session is productive” (92 and 90%, respectively); “Item 16. The therapy session occurs fluently” (85 and 80%, respectively). On the other hand, a clear majority of participants disagree or strongly disagree with the following three statements: “Item 11. I perceive that my client is feeling uncomfortable” (47% for both groups); “Item 17. The session is difficult” (58 and 71%, respectively); “Item 18. The session is shallow” (95 and 88%, respectively). Regarding the impact of wearing a mask on (b) the quality of the verbal and non-verbal communication, for both groups, most of the therapists agree or strongly agree on “Item 2. I can express myself and communicate clearly to the client” (85 and 82%, respectively) and “Item 13. The client can communicate and express his/herself clearly” (81 and 77%, respectively), whereas only a small number of therapists agree or strongly agree on “Item 14. The client has trouble understanding what I am saying” (17 and 11%, respectively). Considering the impact of wearing masks on (c) the quality of therapeutic alliance, for both conditions, the majority of participants agree or strongly agree with the following statements: “Item 4. The explanation and implementation of therapeutic tasks are adequate” (85 and 80%, respectively); “Item 5. The quality of therapeutic work is good” (95 and 86%, respectively); “Item 7. I easily empathize with the client’s experience” (91 and 84%, respectively); “Item 12. “I notice that the client feels safe in the therapeutic relationship” (87 and 76%, respectively). Conversely, a small number of participants agree or strongly agree on “Item 3. Setting goals for the session is hard” (7 and 12%, respectively) and “Item 9. I feel insecure regarding the relationship with my client” (7 and 6%, respectively). For the impact on (d) emotional experience, at least half of participants agree or strongly agree on “Item 6. I can clearly understand the client’s emotional experience” (61 and 57%, regarding existing clients and new clients, respectively) and “Item 15. The client can express his/her emotional experience clearly” (73 and 70%, respectively). Finally, regarding the impact of wearing masks on (e) the safety experience, for both groups, at least half of participants disagree or strongly disagree on “Item 19. I feel at risk of being infected by the new coronavirus (SARS-CoV-2)” (54 and 58%, respectively) and “Item 20. I believe that my client is feeling at risk of being infected by the new coronavirus (SARS-CoV-2)” (63 and 58%, respectively).

### Strategies adopted by therapists in the context of face-to-face therapy wearing a face mask

**FIGURE 3:**
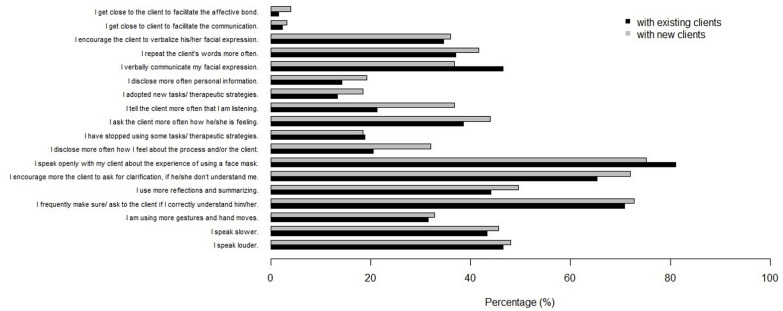
Strategies adopted by psychotherapists.

In considering the strategies the psychotherapists adopted in their face-to-face practice under the COVID-19 pandemic, as can be seen from the data in Figure 3, overall, the percentage breakdown is very similar in considering both conditions–therapy with the existing clients and therapy with the new clients. Thus, the most frequently reported strategy was “I speak openly with my client about the experience of using a face mask,” especially with existing clients, followed by the strategies “I encourage more the client to ask for clarification if he/she doesn’t understand me” and “I frequently make sure/ask the client if I correctly understand him/her,” both a slightly more prevalent with new clients. On the other hand, only a few psychologists reported the need to “get close to the client to facilitate the communication” or “to facilitate the affective bond.”

### How Therapists Experience Face-to-Face Therapy Using Face Masks?

Four main themes resulted from the qualitative analysis: demanding experience; harmlessness and adaptation experience; resources for ensuring efficient therapeutic work; face-to-face therapy as a facilitator of therapeutic work. These themes describe the therapist’s perception of their experience when in face-to-face therapy with a face mask and what they considered significant in this therapeutic context. These themes must be understood as inter-connected at the subordinate level, as participants’ response codes were non-mutually exclusive, and the same participant contributed to different sub-themes or themes at the same time. This implies that the therapists’ experience is not perceived as monothematic but mixed meanings sometimes emerged.

Although these four themes are theoretically meaningful describing different aspects of the therapists’ reported experience, some of them are more prevalent in the data, contributing to a more robust thematic pattern.

Table 1 presents the prevalence of the main themes and subthemes in the two conditions of analysis, therapy with existing clients (TEC) and therapy with new clients (TNC), making it possible to report cross-condition similarities and distinctions. As Table 1 shows there are few differentiating themes/sub-themes from the experience of therapists in the two conditions. Therefore, we refer to therapists’ experiences in general or across both conditions when differences in the respective prevalence occur. Following we describe in detail the “general” and “typical” themes that will be further defined based on their subthemes and illustrated with participants’ quotations. The used code refers to the therapy condition and the ID participant (e.g., TNE_56 refers to the therapist 56 working with a new client).

**TABLE 1:** Prevalence of the main themes and subthemes in the two study conditions.

Main themes and Subthemes	Therapists with existing clients (*N* = 127)	Therapists with new clients (*N* = 124)
**1. Demanding experience**	**General**	**General**
1.1. Uncomfortable experience	Variant	**Typical**
1.2. Difficulties on the therapeutic work	Variant	Variant
1.3. Difficulties in the therapeutic relationship	Rare	Variant
1.4. Difficulties in communication	**Typical**	**Typical**
**2. Harmlessness and adaptation experience**	**Typical**	Variant
2.1. No impact on the therapeutic process	Variant	Rare
2.1. No impact on the therapeutic relationship	Rare	Rare
2.1. Satisfactory experience	Rare	Variant
2.1. Progressive adaptation	Variant	Rare
**3. Resources to ensure efficient therapeutic work**	**General**	**General**
3.1. To take care of the communication	Variant	Variant
3.2. To normalize the face mask use	Variant	Variant
3.3. To take care of the therapeutic relationship	Rare	Variant
3.4. To strengthen the focus on therapeutic skills	Rare	Variant
3.5. To use complementary therapy modalities	Rare	Rare
3.6. To create safety conditions	Rare	Rare
**4. Physical presence as a condition to enable therapy**	**Typical**	**Typical**
4.1. The face mask made the therapy feasible	Variant	Variant
4.2. Face-to-face therapy as a preference	Variant	Variant
4.3. Breaking down barriers found in online therapy	Rare	Rare

#### Theme 1: Demanding Experience

Four subthemes describe the demanding experience reported by both the therapists working with existing clients and therapists working with new clients, showing that therapists’ experience was perceived as uncomfortable, and characterized by difficulties in the therapeutic work, in the therapeutic relationship, and in communication. Next, we describe the different sub-themes and illustrate them in more detail.

*Uncomfortable experience* was typically mentioned by therapists working with new clients. For instance, therapists described their experience as a strange feeling, physical and breathing discomfort, exhausting, or frustrating experience, fears regarding safety. One therapist described her uncomfortable experience as follows: “*It creates an initial discomfort, and it makes the session more tiring*” (TNC_68). Similar uncomfortable experiences were variantly reported by therapists working with existing clients. One therapist described her uncomfortable experience as follows: “*Greater tiredness when there are several consecutive sessions because of the breathing difficulties*” (TEC_23).

*Difficulties on the therapeutic work* were variantly mentioned by therapists in both conditions, who reported the use of face masks as making it difficult to collect clinical information, reading or expressing emotions. In addition, these therapists considered that the face mask sometimes makes it difficult to be attentive to the client and prevents the use of some therapeutic tasks. As reported by two therapists: “*[.]Sometimes managing my own mask interferes with the attention I give to clients*” (TNC_11) and “*I cannot use the mirror technique with the facial expression*” (TEC_51). Beyond these shared difficulties on the therapeutic work, in the case of therapists working with new clients the unfamiliarity with the client’s face was reported as a barrier to the therapeutic work. As indicated by one therapist “*With clients whom we have never seen the face, there may be some emotional expression difficulties*” (TNC_33).

*Difficulties in the therapeutic relationship* were variantly mentioned by therapists working with new clients and rarely mentioned by therapists working with existing clients. In both conditions, therapists reported difficulties on relationships in general or more specifically on empathic rapport or emotional bond. One therapist described this experience saying: “*Difficulty to establish a relationship of trust and empathy, the mask works as a significant obstacle in new cases*” (TNC_77).

*Difficulties in communication* were typically mentioned by therapists whether working with existing or new clients. In both conditions, therapists refer to difficulty in grasping verbal and non-verbal communication, and specifically to clearly understand the facial expression. One therapist refers to these difficulties as follows: “*More difficult because of constraints in communication, either verbal because the client does not perceive us so well or facial because we do not observe the client’s expressions or he ours*” (TEC_38).

#### Theme 2: Harmlessness and Adaptation Experience

Four sub-themes describe the harmlessness and adaptation experience: no impact on the therapeutic relationship, no impact on the therapeutic process, satisfactory experience, and progressive adaptation. These four sub-themes converge to a shared meaning explained by the therapists’ reports about their experience or their attitude regarding the use of face masks in therapy, suggesting that there was no significant impact on therapy. Typically, the therapists working with existing clients referred to this experience, by predominantly reporting no impact on the therapeutic process. The therapists working with new clients only variantly reported this harmlessness and adaptation experience, and predominantly described it as a satisfactory experience.

*No impact on the therapeutic process is a subtheme that* refers to the therapists’ experience or evaluation of the use of mask as not impeding the therapeutic work and therapy process, and the perception that, in general, the therapy occurs as usual. As one of the therapists said: “*In general, the therapy process does not show any change compared to the pre-pandemic*” (TEC_77).

*No impact on the therapeutic relationship is a subtheme that* refers to the therapists’ experience or their evaluation of the use of masks as not creating limitations on the establishment of the therapeutic alliance or therapeutic relationship in general. One therapist reports this experience as follows: “*It is much more tiring, but I do not notice any constraint in terms of the therapeutic bond*” (TEC_119).

*Satisfactory experience* is outlined by therapists’ moderately positive descriptions regarding their therapy experience such as tolerable, safe, acceptable, quiet, comfortable experience and without negative issues. One therapist working with new clients said: “*Everything is going pretty well*” (TNC_50) and another working with familiar clients said: “*It has been running smoothly and with tranquility*” (TEC_163).

*Progressive adaptation* includes therapists’ indications that early discomfort, feelings of strangeness, or difficulties were progressively solved and as well as mentions of a progressive adaptation to the use of the face mask in the therapy context. In this regard, one therapist said: “*It is uncomfortable in the beginning since we are unable to see the entire face and identify the facial expressions so well, but over time this discomfort disappears*” (TNC_54).

#### Theme 3: Resources to Ensure Efficient Therapeutic Work

The third theme connects five sub-themes related to the resources that the therapists used or recommended to guarantee the therapeutic work quality and efficiency, while using a face mask in the context of therapy: to take care of the therapeutic relationship, to take care of the communication, to strengthens the focus on therapeutic skills, to normalize the face mask use and to use complementary therapy modalities. Almost all therapists working with existing clients described different types of resources that they used or recommended to other therapists when using face masks in therapy, while with more emphasis on taking care of the communication and on normalizing the use of masks.

*To take care of the communication* was a resource variantly mentioned by therapists in both conditions who recommended and reported their increased focus on the clarity of verbal and non-verbal behaviors as well as the communication check and feedback. One therapist described this resource as follows: “*Try to speak more slowly and articulate words better to make understanding easier. Check with the client if communication was clear*” (TEC_105).

*To normalize the face mask use* was another resource variantly mentioned by therapists in both conditions. The therapist referred as useful the discussion with the client about the pertinence of using the mask, underlying its provisory use, and accommodating it in the current pandemic situation. One therapist described this resource as follows: “*It is important to discuss with the client the implications of using a mask in the therapeutic process*” (TNC_61) and another therapist said: “*It is only temporary*” (TEC_43).

Almost all the therapists working with new clients referred to resources to ensure the efficient therapeutic work they used by reporting different types. Similarly to therapists working with existing clients, they reported the need to take care of the communication and normalization of masks, but they variantly refer to taking care of therapeutic relationships and to strengthening the focus on the therapeutic skills.

*To take care of the therapeutic relationship* is a sub-theme that includes therapists’ suggestions to take care of the quality of the therapeutic rapport and the therapeutic bond, to involve the client in the resolution of the difficulties, and to encourage the use of empathic skills. One therapist said: “*It’s important to clarify more frequently with the client if he/she is feeling well recognized, understood, and comfortable as well*” (TNC_31).

*To strengthen the focus on therapeutic* skills was variantly mentioned by therapists working with new clients, yet only rarely mentioned by therapists working with existing clients. For instance, therapists talked about reinforcing the use of active listening and interviewing skills, encouraging the verbal expression of emotions, trusting in the therapeutic process, and asking for supervision. As one therapist said: “*Find some alternatives to help the client signal what he is feeling, be aware that you will need more time to understand the client*” (TNC_7).

*To use complementary therapy modalities and to assure health protection* are two sub-themes rarely mentioned by therapists in both conditions. First, a few therapists considered that to complete the information or to become familiar with the client’s face it would be useful to negotiate with the client to complement the therapy with video or email modalities. As said by one therapist “*For the 1st appointment only, I consider video modality more appropriate as we are not wearing a mask, which facilitates communication and the therapeutic relationship*” (TNC_152). To create safety conditions for therapeutic work, a few therapists referred to the need to comply with and reinforce the regular health-protecting measures as well as any specific contextual procedures related to it. For example, one therapist said: “*Take care to disinfect the cabinet, maintain the safety distance and be careful when using the mask*” (TEC_78).

#### Theme 4: Physical Presence as a Condition to Enable Therapy

Three sub-themes describe the fourth theme typically described by therapists in both conditions, illustrating that physical presence was considered as a condition that sometimes is in line with the *clients or therapists’ preferences*, being useful *to break down barriers found in online therapy* and that was *made feasible by the use of face masks*.

*The face mask made the therapy feasible*. Therapists in both conditions variantly mentioned that the face mask contributes to creating a safety climate and protecting clients’ and therapists’ physical health, making it possible to do face-to-face therapy. As one therapist said: “*Honestly, without going overboard, the only positive aspect is, without doubt, the containment of the virus and the promotion of health for patients and psychologists*” (TEC_5).

*Face-to-face therapy as a preference*. Similarly, to the above subtheme, therapists in both conditions variantly mentioned that sometimes face-to-face therapy was a clients’ option corresponding to their preferences, independently of their access to other modalities. In addition, some therapists manifest a similar preference for face-to-face therapy when in comparison with other modalities, as long as the conditions for the protection of health are ensured. For example, one therapist said: “*There would be clients that would give up if therapy was not face-to-face*” (TEC_3).

*Breaking down barriers found in online therapy*. Therapists in both conditions rarely mentioned the advantage of face-to-face therapy when compared with online therapy. They argued based on better conditions for therapeutic relationship development and guarantee of privacy. Some of these therapists also affirmed their belief in the higher efficacy of face-to-face therapy. As reported by one therapist about this subtheme: “*For many clients, face-to-face therapy is more comfortable than online, and it makes it easier to create the therapeutic relationship*” (TNC_105).

**FIGURE 4:**
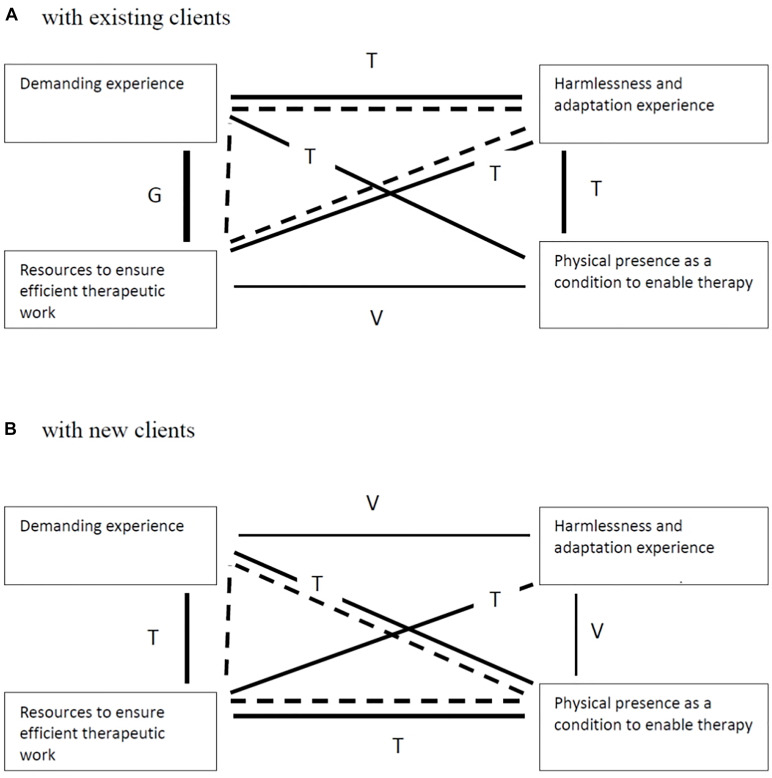
Typical model of therapists’ experiences. Panel **(A)** with existing clients. Panel **(B)** with new clients. G = General link, T = Typical link, V = Variant link. Dashed line represents the typical model.

In sum, these four main themes represent our interpretations grounded on the therapists’ descriptions of their experience and evaluations of face-to-face therapy when using a face mask. As we affirmed above, although representing distinct and coherent patterns of meaning, the main themes are connected at the subordinate level. This connection allows us to describe a trustworthy narrative of the therapists’ experience. Figure 4 shows the links between themes based on their shared prevalence (using the same criteria to decide each theme prevalence) as well as two models describing the typical experience of therapists in both conditions (working with existing or new clients).

As shown in Figures 4A,B, the therapists in both conditions similarly described their experiences, by sharing the meaning patterns involved in two main themes (demanding experience and resources) but also as different in what concerns a third main theme. Typically, therapists working with existing clients described their experience as being demanding but, simultaneously, they also identified several resources to ensure efficient work and considered that the face mask has no negative impact as their experience was described as harmlessness and an adaptation experience (Figure 4A). Although similarly, the therapists working with new clients, typically, described their experience as being demanding and identified several resources to ensure efficient work, they highlighted the physical presence as a condition to enable therapy (Figure 4B).

## Discussion

This mixed study explored the therapists’ experience in face-to-face therapy wearing face masks, differentiating two conditions: working with existing clients and working with new clients, in the context of the COVID-19 pandemic. Through a questionnaire developed for the present study, including both items based on the empirical literature findings and open questions asking for the therapists’ perspectives on their experience, we achieved complementary knowledge on the studied phenomenon.

First and foremost, therapists in both conditions unexpectedly seem not to differ substantially in their evaluation of the impact of wearing a mask on the studied therapeutic dimensions. However, based on the literature review focused on the present study field, we would expect therapists’ more unsatisfactory ratings of the items on these dimensions studied, mainly when therapists referred to the therapeutic work with new clients. Furthermore, these findings are not allied to the previously referred impact of face masks on communication and interpersonal connection quality (Saunders et al., 2020; Vainshelboim, 2020).

Our reflection on these results suggests different possibilities of understanding: on the one hand, given that the study was carried out almost a year after the pandemic breakdown, the therapists’ assessments may have been mediated by the adaptation process to the phenomenon and the consolidation of strategies to solve the difficulties as well, thus minimizing the perceived impact. This possibility seems coherent with the identification of strategies related to the normalization of mask use. Moreover, we hypothesize that a more unfavorable assessment could create cognitive dissonance in therapists who must use the face-to-face intervention modality in the context of the pandemic. Thus, the assessment of the items might have been influenced by the social desirability (e.g., Leary et al., 2015), contributing to reducing the therapists’ cognitive dissonance.

Despite the apparent absence of differences in evaluating the perceived impact of masks, therapists seem to have differentiated the preferred strategies used in each condition. The results show that therapists seem to value more strategies for normalizing or accommodating the mask when working with existing clients while seeming to value strategies focused on communication and therapeutic skills more when working with new clients. Interestingly, these results suggest different therapists’ experiences in both conditions, although we did not observe them as reflected in the perceived impact assessment. In our opinion, it is understandable and even expected that therapists did more attention to communication skills when working with new clients, whose knowledge of facial and emotional expression is more limited. On the other hand, normalization of masks in therapy with existing clients seems to function to accommodate the new circumstance of therapy and respond to other types of needs, such as ensuring confidence in the therapeutic process and its continuity in another format. Indeed, the importance of therapeutic communication quality to achieve and maintain a good quality of therapeutic alliance and the confident dyad engagement is well-documented in previous studies (e.g., Foley and Gentile, 2010; Del Giacco et al., 2020).

Second, the qualitative analysis expands the quantitative results describing the therapists’ experience based on their voices, offering a more detailed and deeper understanding of it. Almost all therapists, whether they refer to their work with existing clients or with new clients, expressed a demanding experience and reported valuable resources to manage the difficulties identified. We would emphasize the typical reference to therapist communication difficulties in both conditions and the discomfort reported typically by therapists working with new clients as contributing to the demanding experience. These results are in line with findings in the literature (e.g., Saunders et al., 2020; Vainshelboim, 2020) and appear to be consistent with the resources reported by the therapists. Note that the therapists reported different strategies in both conditions. However, despite other strategies, those focused on communication and normalization were similarly reported as helpful in both conditions. In addition, it seems that wearing a face mask when working with new clients allows therapists to pay attention to the therapeutic relationship (e.g., difficulties in therapeutic relationship; taking care of the therapeutic relationship). Thus, the therapists’ perspectives add further evidence to the relevance of non-verbal behaviors and facial and emotional expression to the development of therapeutic bond or the empathic negotiation of goals and tasks and expand the previous findings of communication clarity and therapeutic relationship quality (e.g., Foley and Gentile, 2010; Del Giacco et al., 2020).

In this study, the two models that we propose suggest a similarly demanding experience and similar resources or strategies to manage the difficulties encountered. However, two main themes differentiate the therapists’ experience wearing a face mask when working with existing and new clients. First, the therapists expressed more the harmlessness and adaptation experience when working with existing clients, both concerning the therapeutic relationship and the therapeutic work. Second, while working with new clients, therapists expressed the physical presence as a condition to enable therapy. In our view, the distinction between models regarding the therapists’ experience suggests that the previous familiarity and knowledge about the clients favor progressive adaptation and the perception of harmlessness experience. On the other hand, when therapists and clients are not mutually well-known, physical presence seems to be an essential condition for the viability of therapy, whether in articulating the therapy at a distance and despite the constraints generated by the use of the mask.

In sum, the apparent perception of the harmlessness of the face mask in therapy seems to be a consistent result of cross-quantitative and qualitative analysis. However, the qualitative study allowed us to perceive in detail and depth the demanding experience, the adaptation process, and strategies that allow the therapists to overcome the constraints placed by the face mask and explain the challenges posed by the situation. Considering almost all participants’ practice-based knowledge and experience, which grounded the models we present in this study, we can formulate some tempting recommendations that seem to enhance the efficacy and quality of the therapeutic work. Namely, with existing clients, we highlight the relevance of the therapist normalizing face masks wearing through the discussion of implications of using it. Concerning the new clients, we suggest focusing on verbal and non-verbal communication skills, clarifying any constraints in communication as much as needed.

### Strengthens and Limitations

The study sample seems to be sufficiently large. Furthermore, its demographic characteristics are similar to those published on the last census performed by Ordem dos Psicólogos Portugueses (2014), indicating that participants in this study are probably representative of the broader clinical practitioner body. Additionally, we believe this exploratory study provides exciting and in-depth information based on psychotherapists’ experiences. It allows us to expand our understanding, adjust and improve face-to-face practice. Due to pandemic restrictions imposed by lockdown to the research team (mobility to the research lab and using the same computer/equipment), it was impossible to have access to the Nvivo Software, which is appropriate and one of the most used tools for qualitative analysis. Alternatively, we used Excel software to perform the qualitative analysis, which is a very accessible and flexible tool. However, the analytic procedure was more time-consuming and demanding in terms of data organization.

Nevertheless, this study targeted psychotherapists’ experiences of face mask-wearing under face-to-face therapeutic processes only with new or existing clients. The purposive sampling used in the current exploratory study might have limited the generalizability of the obtained results. However, as most participants pertain to both conditions (existing and new clients), the bias may have been attenuated.

### Future Studies

Regarding future studies aiming to expand the current study findings, we suggest different research possibilities. First, we recommend replicating the present study considering clients‘ experiences during face-to-face therapy wearing a face mask to understand what might be distinct or similar to the therapists’ experiences. Second, we suggest implementing a qualitative interview-based study using contrasting groups (i.e., therapists that only attend new clients/therapists that only treat existing clients), aiming to consolidate the models we developed about therapists’ experiences. Furthermore, based on non-verbal communication’s role in psychotherapeutic relational and emotional processes, we suggest future studies to compare the impact of barriers to communication in different psychotherapy conditions, such as face-to-face therapy wearing face masks and online therapy (phone call, video call, and email). In addition, we are aware that cultural factors (languages, beliefs, and values) can affect what and how we communicate and how we behave, so in further studies, it could be essential to understand and consider the influence of cultural factors on the relational and communicational processes in psychotherapy.

## Data Availability Statement

The raw data supporting the conclusions of this article will be made available by the authors, without undue reservation.

## Ethics Statement

The studies involving human participants were reviewed and approved by Comissão de Ética para a Investigação em Ciências Sociais e Humanas (CEICSH); Universidade do Minho. The patients/participants provided their written informed consent to participate in this study.

## Author Contributions

ER, ÂF, CC, RQ, and VS contributed to the conception and design of the study. ER, CC, and VS wrote the introduction section. ÂF and VS organized the database. ÂF performed the statistical analysis. CC, RQ, VS, and ER performed the qualitative analysis. ER and ÂF wrote the materials and methods and results sections, ER, ÂF, and RQ wrote the discussion section of the manuscript. All authors contributed to manuscript revision, read, and approved the submitted version.

## Conflict of Interest

The authors declare that the research was conducted in the absence of any commercial or financial relationships that could be construed as a potential conflict of interest.

## Publisher’s Note

All claims expressed in this article are solely those of the authors and do not necessarily represent those of their affiliated organizations, or those of the publisher, the editors and the reviewers. Any product that may be evaluated in this article, or claim that may be made by its manufacturer, is not guaranteed or endorsed by the publisher.
